# Oral Anticoagulants Preference in Hospitalized Patients with Acute Deep Vein Thrombosis or Non-Valvular Atrial Fibrillation

**DOI:** 10.3390/healthcare8040404

**Published:** 2020-10-15

**Authors:** Ştefan Cristian Vesa, Sonia Irina Vlaicu, Sorin Crișan, Octavia Sabin, George Saraci, Vitalie Văcăraș, Daciana Elena Popa, Paula Pârcălab, Valer Ioan Donca, Antonia Eugenia Macarie, Madalina Sava, Anca Dana Buzoianu

**Affiliations:** 1 Department of Pharmacology, Toxicology and Clinical Pharmacology, “Iuliu Haţieganu” University of Medicine and Pharmacy, 400337 Cluj-Napoca, Romania; stefanvesa@gmail.com (S.C.V.); abuzoianu@umfcluj.ro (A.D.B.); 2 Department of Internal Medicine, 1st Medical Clinic, “Iuliu Haţieganu” University of Medicine and Pharmacy, 400006 Cluj-Napoca, Romania; vlaicus@yahoo.com; 3 Department of Internal Medicine, 5th Medical Clinic, “Iuliu Haţieganu” University of Medicine and Pharmacy, 400139 Cluj-Napoca, Romania; crisan.sorin@gmail.com; 4 Graduate of “Iuliu Haţieganu” Faculty of Medicine, University of Medicine and Pharmacy, 400337 Cluj-Napoca, Romania; 5 Department of Neurology, “Iuliu Hațieganu” University of Medicine and Pharmacy, 400012 Cluj-Napoca, Romania; vvacaras@umfcluj.ro; 6 Department of Cardiology, “Niculae Stăncioiu” Heart Institute, 400001 Cluj-Napoca, Romania; dacianaelenapopa@gmail.com; 7 “Prof. Dr. Ion Chiricuță” Oncology Institute, 400010 Cluj-Napoca, Romania; paulagparcalab@gmail.com; 8 Department of Geriatrics-Gerontology, “Iuliu Haţieganu” University of Medicine and Pharmacy, 400139 Cluj-Napoca, Romania; valerdonca@gmail.com (V.I.D.); macarieantonia@yahoo.com (A.E.M.); 9 Department of Dermatology, Emergency County Hospital, 410032 Oradea, Romania; madalina.sava0508@gmail.com

**Keywords:** atrial fibrillation, deep vein thrombosis, anticoagulant choice, direct oral anticoagulants, vitamin K antagonists

## Abstract

(1) Aim: The aim of this study was to assess the preferences of oral anticoagulants (OA) in patients diagnosed with deep vein thrombosis (DVT) of lower limbs or non-valvular atrial fibrillation (AF) requiring anticoagulation for medium/long term. (2) Materials and methods: the study included consecutive patients admitted with a diagnosis of either acute DVT of lower limbs (without signs of pulmonary embolism) or non-valvular AF who required oral anticoagulation, in a time frame of 18 months from January 2017 until June 2018. The following data were recorded: demographic variables, comorbidities (ischemic heart disease, arterial hypertension, heart failure, stroke, peripheral artery disease, diabetes mellitus, obesity), type and dose of OA (acenocoumarol, dabigatran, apixaban, rivaroxaban), complications due to the use of OA. (3) Results: AF patients were older and had considerably more cardiovascular comorbidities than DVT patients. Vitamin K antagonists (VKA) were more likely to be administered in patients with AF, as they had indication for indefinite anticoagulation. VKA were more frequently prescribed in patients with ischemic heart disease, heart failure, and diabetes compared with DVT patients. Moreover, complications related to OA use were more frequent in the VKA group. Almost half of patients with acute DVT (48.5%) were treated with direct OA (DOAC) rather than VKA, and only a quarter of AF patients (24.8%) were treated with DOACs.

## 1. Introduction

Hypercoagulable state is an important issue in modern medicine, relevant to every clinical specialty including visceral, obstetrical, and orthopedic surgery or medical branches like cardiology, pulmonology, gastroenterology, oncology, internal medicine, and recently described COVID 19. The main triad of clot formation was depicted more than 100 years ago by Rudolf Virchow and includes hypercoagulation, parietal lesion, and blood stasis, and these aspects are ubiquitous in every case of thrombus formation [1]. From that moment, a wide range of therapeutic approaches have been implemented to date, the most important one being the advent of the new oral anticoagulant era, more than ten years ago. From their first use in orthopedic surgery, especially in knee surgery, use of these drugs expanded across the field year after year in many other conditions like atrial fibrillation, deep vein thrombosis, and malignancies [2].

Atrial fibrillation (AF) is a clinical entity characterized by the loss of sinoatrial node control over atrial depolarization and contraction and its replacement with chaotic disorganized electric and mechanical activity of atria, with subsequently compromised pomp function and disturbances also in their conduct and reservoir functions [3]. Even with the availability of recently introduced procedures like radiofrequency ablation or left atrial appendage closure devices, anticoagulant therapy remains mandatory after these device procedures as thrombi can originate also in the left atrium [4]. The necessity of anticoagulation in AF is established by the CHA2Ds2-VASc score and the hemorrhagic risk is estimated by HAS-BLED score [5]. As studies the showed noninferiority of direct oral anticoagulants (DOAC) when compared to vitamin K antagonists (VKA) with the benefit of lower hemorrhagic events, choosing the oral anticoagulant (OA) involves evaluating the cost, the accessibility to monitoring INR (International Normalized Ratio), and the risk of having fluctuant INR due to patient characteristics or drug interactions [6].

Deep vein thrombosis (DVT) and pulmonary embolism are initiated by the formation of a thrombus in a deep vein followed by its virtual wedging into distant vessels, mainly branches of pulmonary arteries in the absence of communication between left and right heart. In the latter case, any location within the circulatory system is virtually possible [7]. While for AF, the use of anticoagulation is subject to debate according to previously mentioned scores, in the case of acute DVT, anticoagulant therapy is mandatory and must be continued for a medium or long period after the acute phase, and in certain conditions, its use is lifelong [7].

Choosing between VKA and DOAC for medium/long term is problematic, as acquiring the target INR could be difficult for some patients and is a reason for multiple ambulatory consultations. There is no consensus on this issue, so the physician must discuss and establish the adequate product for each patient. Besides cost, the only shortcoming of DOAC—until recently—was the lack of an antidote in the case of overdose or high serum levels of various causes; this obstacle was overcame with the introduction of Idarucizumab for dabigatran and Adexanet alpha for direct factor Xa inhibitors, thus making the choice even more complicated as the new antidotes are expensive [8].

The aim of this study was to assess the preferences of oral anticoagulant (OA) in patients diagnosed with DVT of lower limbs or non-valvular AF, who required anticoagulation for medium/long term.

## 2. Materials and Methods

The study was a retrospective, analytic, observational, transversal, cohort type. We included all consecutive patients admitted with a diagnosis of acute DVT of lower limbs without signs of pulmonary embolism or non-valvular AF, who required oral anticoagulation, in a time frame of 18 months from January 2017 until June 2018. The study was approved by the hospital’s ethics committee (no. 350/13 November 2014) and all patients gave informed consent. Patients diagnosed with cancer or autoimmune diseases were excluded from the study.

The following data were recorded: demographic variables, comorbidities (ischemic heart disease, arterial hypertension, heart failure, stroke, peripheral artery disease, diabetes mellitus, obesity), type and dose of OA (acenocoumarol, dabigatran, apixaban, rivaroxaban), complications due to OA (upper or lower gastrointestinal bleeding, hematuria, epistaxis, ecchymosis, INR ã 4.5).

A statistical analysis was carried out using the MedCalc Statistical Software version 19.4.1 (MedCalc Software bv, Ostend, Belgium, 2020). The normality of distribution for the quantitative data was verified with the Shapiro–Wilk test. Quantitative variables were described using mean and standard deviation. Qualitative data were characterized by frequencies and percentages. Between-group comparisons were performed using the Student T test for quantitative variables and chi-square tests or Fisher’s exact test for qualitative variables. A multivariate analysis was employed to determine which variables were independently associated with OA preference. Variables that reached statistical significance in the univariate analysis were introduced in a logistic regression. A *p* value > 0.05 was considered statistically significant.

## 3. Results

Patients’ characteristics are summarized in Table 1. Most of the patients with AF had advanced age and presented statistically significant more cardiovascular comorbidities, as compared with patients with DVT.

**Figure 1:**
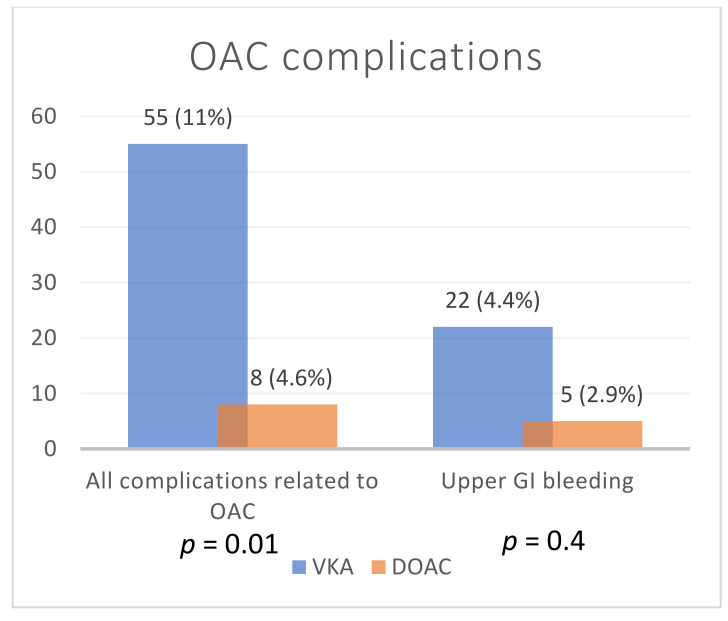
Oral anticoagulant complications. OAC—oral anticoagulants; VKA—vitamin K antagonist; DOAC—direct oral anticoagulant; GI—gastro-intestinal.

There were several differences between patients treated with VKA and those receiving DOAC (Table 2). VKA were more frequently prescribed in patients with ischemic heart disease, heart failure, and diabetes. Complications related to OA use were more frequent in the VKA group (Figure 1).

A multivariate logistic regression was used to determine the independent association of several variables with OAC preference (Table 3). Patients with atrial fibrillation, ischemic heart disease, or diabetes mellitus had a higher probability of receiving VKA.

**Figure 2:**
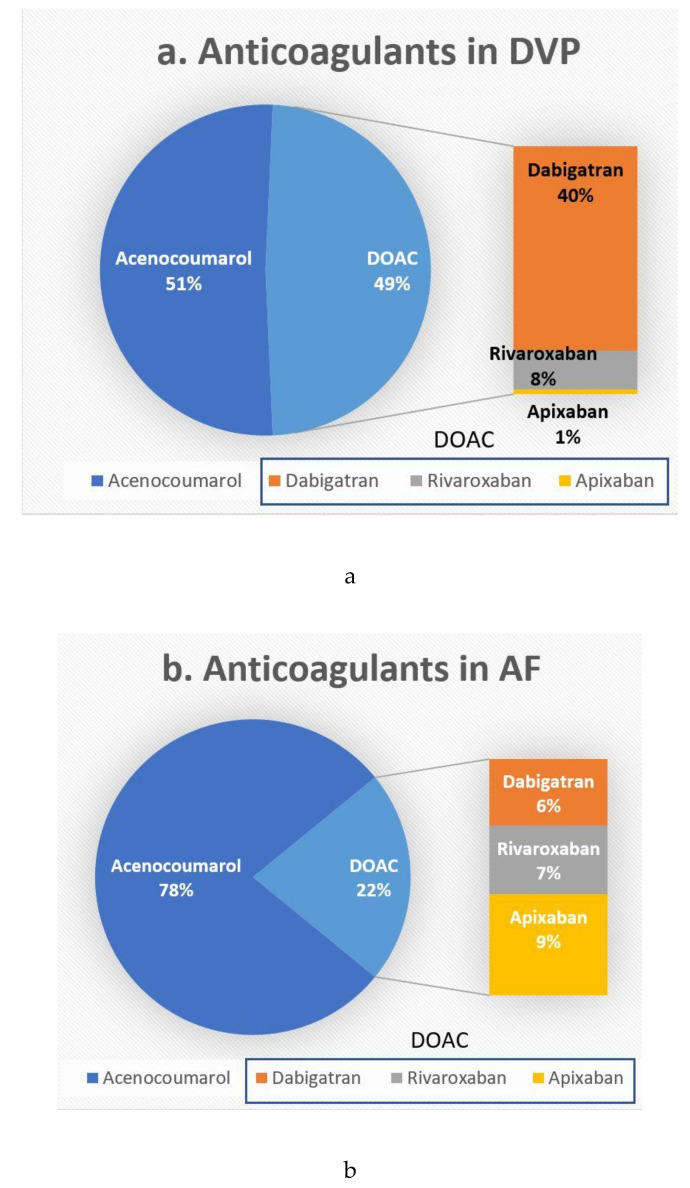
Anticoagulant choice. DOAC—direct oral anticoagulant (**a**). DVP—deep vein thrombosis (**b**). AF—atrial fibrillation.

Dabigatran was administered to 77 patients; 41 (53.2%) of them received 150 mg p.o b.i.d. and 36 (46.7%) received 110 mg p.o b.i.d. Rivaroxaban was administered to 44 patients, from which seven (15.9%) received 15 mg p.o b.i.d., 20 (45.4%) received 20 mg p.o q.d., and 17 (38.6%) received 15 mg mg p.o q.d. Apixaban was administered to 54 patients, from which 41 (75.9%) received 2.5 mg p.o b.i.d., and 13 (24.1%) received 5 mg p.o b.i.d. Acenocoumarol was administered to 499 patients; 68 (13.6%) of the acenocumarol treated patients received over 28 mg weekly, 277 (55.5%) received between 7 and 28 mg weekly, and 154 (30.8%) received under 7 mg weekly. Frequencies of use for each class of anticoagulants are detailed in Figure 2a,b.

## 4. Discussion

In the last decade, DOACs have secured a leading place as anticoagulant treatment in several diseases. They are safer drugs, with an efficiency at least equal to VKA, which does not require strict therapeutic monitoring [5,7,9]. Initially indicated in venous thromboembolism (VTE) prevention, later in stroke prevention in AF, DAOCs have been shown to be effective in the treatment of acute DVT, replacing conventional therapy, heparins, and VKA in several patients [9,10]. From the beginning of their use, two major drawbacks were noticed: the much higher price and the lack of an antidote in case of overdose. Attempts to overcome these drawbacks consist in the gradual reduction of costs, the introduction of reimbursement of these drugs in the insurance systems, the approval of generic variants of some of these drugs (rivaroxaban), and reversal agents. The medical community has witnessed a continuously growing interest in the last few years in replacing “the old coumarins” to these new “easy-to-take” anticoagulants.

A gradual increase in the proportion of DOAC usage in AF treatment was recently documented all over the world [11,12,13]. DOACs are initiated more frequently than VKA in numerous countries [11,13,14], contributing to an increase in appropriate prescribing of anticoagulants in AF. Despite DOACs’ current large availability and the increasing adherence to anticoagulation guidelines, sub-optimal anticoagulation in AF [13,15] and inappropriate use at DOAC initiation still represent pertinent issues [16].

The shift from VKA to DOACs was also observed for acute DVT treatment, after several studies showed that they constitute an effective and safer alternative to conventional anticoagulation [17]. There are no head-to-head trials to date to compare DOACs, but meta-analysis research has reported that DOACs are equally effective in preventing strokes and systemic embolisms in AF [9] and acute DVT [10]. Apixaban treatment results in a significant reduction in major bleeding and clinically relevant bleeding compared to other DOACs [9,10]. Therefore, guidelines now prefer DOACs over conventional treatment for acute DVT treatment in patients without active cancer [7]. Current recommendations in acute DVT treatment do not discriminate between DOACs and indicate that dabigatran, endoxaban, and VKA treatment should be preceded by at least 5 days of low weight molecular heparins. As for apixaban and rivaroxaban, it is possible to begin an oral treatment with these DOACs from day 1. The aforementioned arguments may seem as a huge advantage in theory, but in real-life practice, the reluctance of some doctors with a lifelong experience with VKA treatment (“the classic way”) can be a significant obstacle in choosing DOACs.

In our study, AF patients were older and had considerably more cardiovascular comorbidities than DVT patients. VKA were more frequently prescribed in patients with ischemic heart disease, heart failure, and diabetes compared with DVT patients. Moreover, complications related to OA use were more frequent in the VKA group. Patients with comorbidities are more likely to be treated by VKA.

Almost half of patients with acute DVT (48.5%) were treated with DOACs rather than VKA, and only a quarter of AF patients (24.8%) were treated with DOACs. In other similar studies on hospitalized patients with AF, the proportion of DOAC use was higher, from 35% in another hospital in Romania [18] to more than 50% in Greece [19]. Compelling data prove that DOACs are now the preferred therapy for initiation of OA in patients with AF, including in patients at higher risk of stroke or bleeding [11,13]. VKA were more likely to be administered in patients with AF, as they had indication for indefinite anticoagulation. This might be considered a potential major problem if we only consider the risk of bleeding during treatment, which is higher in the case of AF due to the indefinite duration of treatment compared to DVT treatment. In our study, the preference for VKA was probably due to the cost involved: DOACs’ financial burden is sustainable for the patients for 3 to 6 months, but not for a long period. The fact that, at the time of the study (2018), DOACs were not reimbursed by the National Assurance argues for the disproportionate costs of the two OAs being a relevant factor influencing therapeutic decision.

Notably, we have reported a clear predilection for choosing dabigatran in acute DVT (more than 40% patients) over other DOACs. This was not the case for AF patients treated with DOACs, for whom the distribution between the three DOACs available seems equal, with small preference for apixaban (9.3% vs. 6.1% dabigatran and 6.3% rivaroxaban).

The preferences and the local standard practices for DOACs in acute DVT vary worldwide and are influenced by cost, differences in reimbursement, or accessibility of therapies [20]. In the GARFIELD-VTE study, a large multinational observational real-world treatment practice in acute venous thromboembolism, overall, one half of the patients received a DOAC either alone or with transition from parenteral anticoagulant, with a larger proportion in Europe and North America (up to 60%). The most frequently prescribed was rivaroxaban and only a minority, around 5%, received dabigatran [20]. The choice of rivaroxaban was probably the result of it being the first DOAC recommended for the treatment of VTE and the early introduction of generic alternatives. Apixaban is preferred in some countries for AF and VTE [11,16]. Another possible influence over the therapeutic decision is the estimated duration of OA treatment. An estimate of one third [7] of the DVT patients will need a long-term anticoagulation plan, but, unfortunately, there are no follow-up studies for these patients on DOACs vs. VKA over a longer period than one year. Dabigatran was the first DOAC available in Romania and the high percentage of prescription in acute DVT may be explained by physicians’ confidence and experience with this drug [18] and by a slight difference in cost between DOACs in favor of dabigatran. Even if patients with AF have more comorbidities and their anticoagulant treatment is needed indefinitely—which translates into a higher risk of bleeding complications—the clinicians in our center continue to opt for VKA, probably because of economic reasons.

Among the limitations of the study are the lack of information about the socio-economic status of the patients and the lack of follow-up, which is important in the assessment of the rate of complications and in light of the fact that several patients will abandon the treatment with DOACs. A future direction of research would be to evaluate the dynamics in time of the choice of OA, how the costs of these drugs influence the prescription, and physicians’ reasons to choose a particular OA, beside guidelines and drug labeling.

## 5. Conclusions

Our study showed that VKA are the preferred OA for long-term stroke prevention in AF and DOACs are mostly used in patients with DVT—a pathology that requires a shorter period of anticoagulation. Complications related to OA use were more frequent in patients treated with VKA.

## Figures and Tables

**Table 1:** Patients’ characteristics.

Variables		AF (*n* = 569)	DVT (*n* = 105)	*p*
Age (years)		74.6 ± 9.6	65.6 ± 14.7	<0.001
Gender	Female	295 (51.8%)	50 (47.6%)	0.4
	Male	274 (48.2%)	55 (52.4%)	
Ischemic heart disease		390 (68.5%)	25 (23.8%)	<0.001
Arterial hypertension		477 (83.8%)	70 (66.7%)	<0.001
Diabetes mellitus		149 (26.4%)	16 (15.2%)	0.01
Heart failure		467 (82.1%)	28 (26.7%)	<0.001
History of stroke		97 (17.1%)	4 (3.8%)	<0.001
Peripheral artery disease		36 (6.3%)	1 (1%)	0.04
Obesity		136 (23.9%)	42 (40%)	0.5
Chronic kidney disease		134 (23.6%)	11 (10.5%)	0.004
DOAC	Dabigatran	35 (6.15%)	42 (40%)	<0.001
	Rivaroxaban	36 (6.3%)	8 (7.6%)	
	Apixaban	53 (9.3%)	1 (0.9%)	
Acenocoumarol		445 (78.2%)	54 (52.4%)	

AF—atrial fibrillation; DVT—deep vein thrombosis; DOAC—direct oral anticoagulant.

**Table 2:** Comparison between VKA and DOAC group.

Variables		VKA (499)	DOAC (175)	*p*
Condition requiring OAC	AF	445 (89.2%)	124 (70.9%)	<0.001
	DVT	54 (10.8%)	51 (29.1%)	
Gender	Female	260 (52.1%)	85 (48.6%)	0.4
	Male	239 (47.9%)	90 (51.4%)	
Age (years)		73.7 ± 10.1	71.6 ± 13.3	0.06
Ischemic heart disease		333 (66.7%)	82 (44.9%)	<0.001
Arterial hypertension		408 (81.8%)	139 (79.4%)	0.5
Diabetes mellitus		137 (27.7%)	28 (16%)	0.003
Heart failure		381 (76.4%)	114 (65.1%)	0.005
History of stroke		68 (13.7%)	33 (18.9%)	0.1
Peripheral artery disease		30 (6%)	7 (4%)	0.4
Obesity		135 (27.1%)	43 (24.6%)	0.5
Chronic kidney disease		109 (21.8%)	36 (20.6%)	0.8
All complications related to OAC		55 (11%)	8 (4.6%)	0.01
Complications	Upper GI bleeding	22 (4.4%)	5 (2.9%)	0.09
	Hematuria	12 (2.4%)	2 (1.1%)	
	Ecchymosis	10 (2%)	-	
	Epistaxis	11 (2.2%)	1 (0.6%)	

VKA—vitamin K antagonists; DOAC—direct oral anticoagulant; AF—atrial fibrillation; DVT—deep vein thrombosis; OAC—oral anticoagulant.

**Table 3:** Multivariate logistic regression for OAC preference.

Variables	B	*p*	OR	95%CI for OR	
				Min	Max
Age	0.002	0.7	1.002	0.986	1.019
Atrial fibrillation	0.968	<0.001	2.633	1.564	4.433
Ischemic heart disease	0.532	0.008	1.702	1.149	2.521
Diabetes mellitus	0.561	0.01	1.753	1.104	2.782
Heart failure	−0.078	0.7	0.925	0.581	1.472
Constant	0.718	0.235	2.049		

B—regression coefficient; OR—odds ratio; CI—confidence interval.
